# Revolutionizing Cancer Treatment: The Promising Horizon of Zein Nanosystems

**DOI:** 10.1021/acsbiomaterials.3c01540

**Published:** 2024-03-01

**Authors:** Subham Preetam, Deb Duhita Mondal, Nobendu Mukerjee, Shaikh Sheeran Naser, Tanveer A. Tabish, Nanasaheb Thorat

**Affiliations:** † Department of Robotics and Mechatronics Engineering, Daegu Gyeongbuk Institute of Science and Technology, Daegu 42988, South Korea; ‡ Department of Biotechnology, Heritage Institute of Technology, Kolkata, West Bengal 700107, India; § Centre for Global Health Research, Saveetha Medical College and Hospital, Chennai 602105, India; ∥ Department of Science and Engineering, Novel Global Community and Educational Foundation, Hebasham 2770, NSW, Australia; ⊥ KSBT, Kalinga Institute of Industrial Technology, Bhubaneswar 751024, India; # Division of Cardiovascular Medicine, Radcliffe Department of Medicine, University of Oxford, Oxford, OX3 7BN, United Kingdom; ∇ Nuffield Department of Women’s & Reproductive Health, Medical Science Division, John Radcliffe Hospital University of Oxford, Oxford, OX3 9DU, United Kingdom; % Department of Physics, Bernal Institute and Limerick Digital Cancer Research Centre (LDCRC), University of Limerick, Castletroy, Limerick V94T9PX, Ireland

**Keywords:** zein nanoparticles, anticancer therapy, drug delivery system, precision medicine

## Abstract

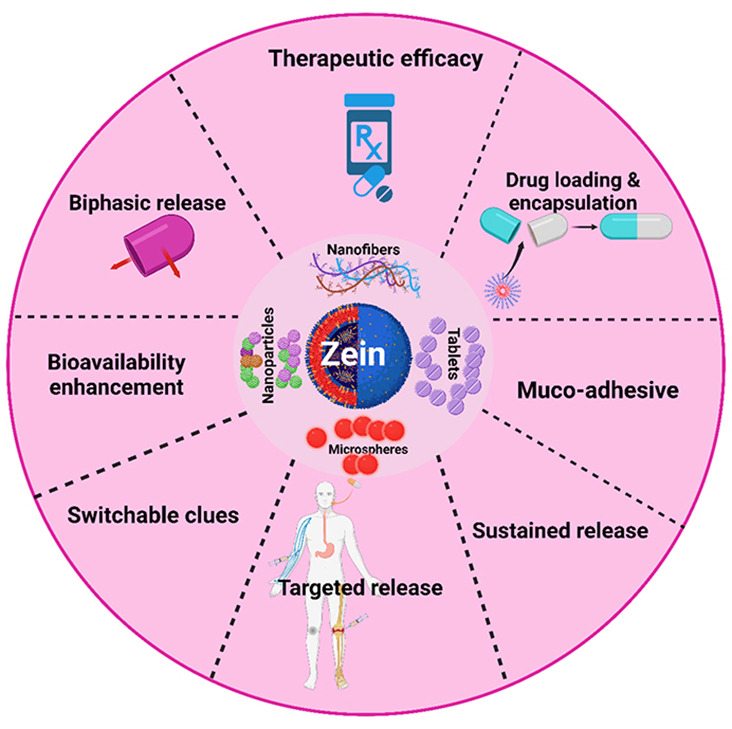

Various nanomaterials have recently become fascinating tools in cancer diagnostic applications because of their multifunctional and inherent molecular characteristics that support efficient diagnosis and image-guided therapy. Zein nanoparticles are a protein derived from maize. It belongs to the class of prolamins possessing a spherical structure with conformational properties similar to those of conventional globular proteins like ribonuclease and insulin. Zein nanoparticles have gained massive interest over the past couple of years owing to their natural hydrophilicity, ease of functionalization, biodegradability, and biocompatibility, thereby improving oral bioavailability, nanoparticle targeting, and prolonged drug administration. Thus, zein nanoparticles are becoming a promising candidate for precision cancer drug delivery. This review highlights the clinical significance of applying zein nanosystems for cancer theragnostic—moreover, the role of zein nanosystems for cancer drug delivery, anticancer agents, and gene therapy. Finally, the difficulties and potential uses of these NPs in cancer treatment and detection are discussed. This review will pave the way for researchers to develop theranostic strategies for precision medicine utilizing zein nanosystems.

## 1.0 Introduction

Drug delivery has significantly improved over the past few years, owing to nanotechnology advancements. The lack of localized and targeted delivery and controlled release of classical chemotherapeutic drugs have been a primary challenge in treating cancer.^1−5^ Nanoparticle fabrication technologies have several benefits, including improving drug solubility and dissolution, shielding therapeutic components from degradation, regulating drug release, extending blood circulation, and boosting accumulation at target areas.^6,7^ Moreover, the efficiency of these versatile drug delivery carriers is enhanced with further engineering of their surfaces to avoid adverse effects.^8,9^ Nanoparticles (NPs) offer distinct structural characteristics, including their shape, size, and surface topologies, which decide their long-term fate in achieving their goal of reaching the site of action.^10−12^ The enhanced permeability and retention (EPR) effect facilitating cancer treatment requires the NPS to be sized in 10–100 nm. The minimal size permits a feasible outlet from the constricted vesicular walls and further filtration by the kidneys without degrading the normal cells by its cytotoxic effects, whereas enlarged surfaces are likely to be discarded via circulation by the phagocytes.^13−15^ The hydrophilicity of coating materials augments the period of drug circulation. It elevates secretion and assembly in tumors, such as in the case of polyethene glycol (PEG) coated molecules inhabiting resistance against the immunogenic response.^16−19^

**Figure 1:**
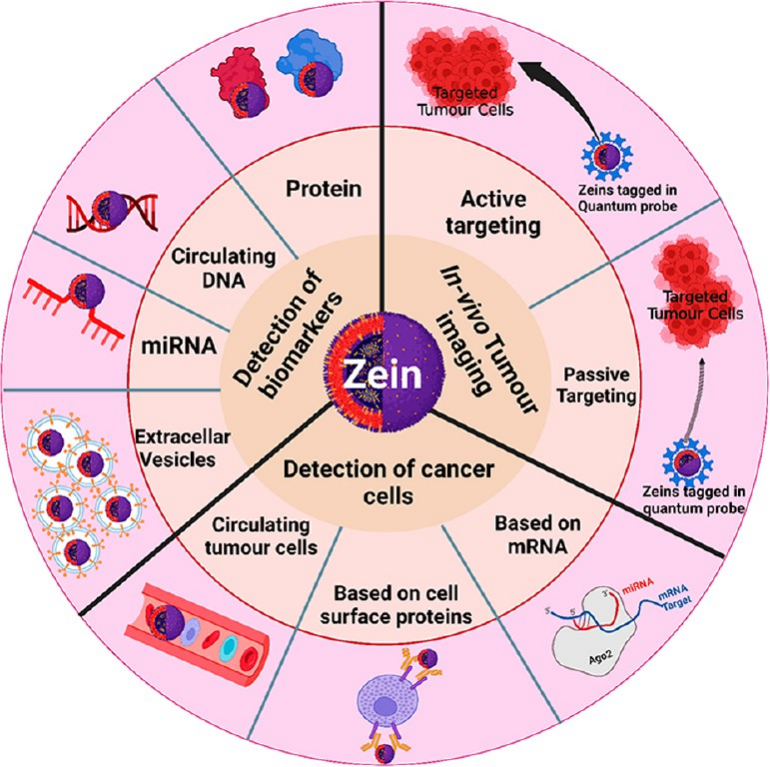
Schematics of zein-nanoparticle-based cancer drug delivery. The figure not only depicts the diverse modes of targeting cancer cells for precision delivery in the tumor microenvironment but also elucidates the theragnostic application for cancer management.

While empirical methodologies and site-specific ligands are pivotal tools for site-specific delivery and are utilized for conventional NP production methods, the optimization strategies for exploiting receptor–ligand interaction are still a matter of study.^18,20−22^ Biomaterials have been extensively utilized in recent years for their picking ability for drug delivery.^23−26^ Proteins derived from animal and plant sources have shown promise in creating NPs with good efficacy, high loading capacity, safety, and functional characteristics.^27−32^ Zein NP is one of them. Due to its inherent hydrophobicity, zein NP has attracted a lot of attention in recent years for its tunability, biodegradability, and biocompatibility, thus causing increased oral bioavailability, prolonged drug administration, and NP targeting (Figure 1). Meanwhile, the creation of NPs can be achieved by repeating spherical blocks of zein. Zein NPs, being insoluble in aqueous environments at a pH of 11, cause precipitation and aggregation in a colloidal system.^33^ As a result, functionalization or alteration of the zein structure intends to make NP generation and targeting easier for drug delivery applications. Several solutions have been proposed to overcome the limitation of zein NPs in recent years (Figure 1), for instance, polymer blended with zein NPs, polymer-based complexes for aiding the delivery of zein NPs, core or shell-based coating for the extended circulatory time of zein NPs, cross-linked zein NPs, and nanofiber-based coating of zein NPs.^34−39^

The background of zein nanosystems is rooted in its biocompatibility and biodegradability, making it an ideal candidate for pharmaceutical use.^40,41^ Its unique properties allow for the encapsulation of a wide range of substances, including both hydrophilic and hydrophobic drugs, which is pivotal in the synthesis and preparation of targeted drug delivery systems.^42^ Current research in this area is focused on exploiting these properties to enhance the efficacy of anticancer agents.^33^ By encapsulating these agents within zein nanoparticles, researchers aim to achieve targeted delivery to cancer cells, thereby increasing the drug’s therapeutic efficiency while minimizing side effects.^38,43^ This targeted approach is crucial in oncology, as it allows for the direct attack on cancer cells while sparing healthy tissues, leading to better patient outcomes.^36,37,44−46^ Additionally, zein nanosystems are being explored in the realm of gene therapy. The ability of these nanoparticles to protect and deliver genetic material into specific cells presents a promising avenue for treating a variety of genetic disorders. This application is particularly groundbreaking, as it opens up possibilities for personalized medicine and treatments for diseases that were previously thought to be untreatable.^19,39,47^ The synthesis and preparation of zein nanosystems involve advanced techniques to ensure the stability, controlled release, and specific targeting of the encapsulated substances. Innovations in this field continue to evolve, with ongoing research aiming to overcome challenges such as scalability, long-term stability, and the precise control of drug release rates.^48,49^ As research progresses, zein nanosystems hold the potential to revolutionize the landscape of drug delivery and gene therapy, offering more effective, safer, and personalized treatment options for a variety of diseases, particularly cancer.^50^

The significance and advantages of zein nanoparticles (NPs) in the realms of anticancer agents and gene therapy are marked by their unique strengths, setting them apart from other nanoparticles.^46,51−53^ One of the primary advantages is their biocompatibility and biodegradability, derived from zein being a naturally occurring protein in corn. This makes zein NPs less toxic and more acceptable to the human body compared with synthetic nanoparticles, thus reducing the risk of adverse reactions during cancer treatment and gene therapy. In the context of anticancer agents, zein NPs offer enhanced targeting capabilities.^54,55^ Their surface can be easily modified to attach specific ligands, allowing for the targeted delivery of drugs to cancer cells while sparing healthy tissue. This targeted approach not only increases the efficacy of the drug but also significantly reduces the side effects commonly associated with traditional chemotherapy.^56^ Furthermore, zein NPs have a unique ability to efficiently encapsulate both hydrophilic and hydrophobic drugs, an attribute not universally present in other nanoparticles. This versatility in drug encapsulation expands the range of anticancer agents that can be delivered using zein NPs, providing flexibility in treatment options.^57−59^

In gene therapy, the nonviral nature of zein NPs presents a distinct advantage. Unlike viral vectors, they do not induce strong immune responses or carry the risk of integrating harmful genetic material into the host genome.^60,61^ This safety profile is crucial, as it minimizes potential complications and broadens the applicability of gene therapies to a wider range of patients. Additionally, the controlled release properties of zein NPs are pivotal. They can be engineered to release their genetic payload or drugs over a specified period, allowing for sustained therapeutic effect and reduced frequency of dosing.^62^ This not only improves patient compliance but also maintains a steady therapeutic concentration, vital for the effective management of cancer and genetic disorders.^39,63^ Overall, the unique strengths of zein NPs—such as their biocompatibility, targeted drug delivery, versatility in encapsulation, nonviral nature for gene therapy, and controlled release capabilities—underscore their significant potential in revolutionizing the treatment of cancer and the delivery of gene therapies, going beyond the capabilities of traditional nanoparticles.^50,64,65^

In this review, we discuss the synthesis, structure, and characterization of zein NPs and their modification with various ligands that benefits their ability to deliver drugs for cancer therapeutics in a safe and targeted fashion. We have emphasized the necessity of these theranostics based on zein NPs for cancer medication resistance.

## 2.0 Characterization, Structure, and Properties of Zein Nanoparticles

**Figure 2:**
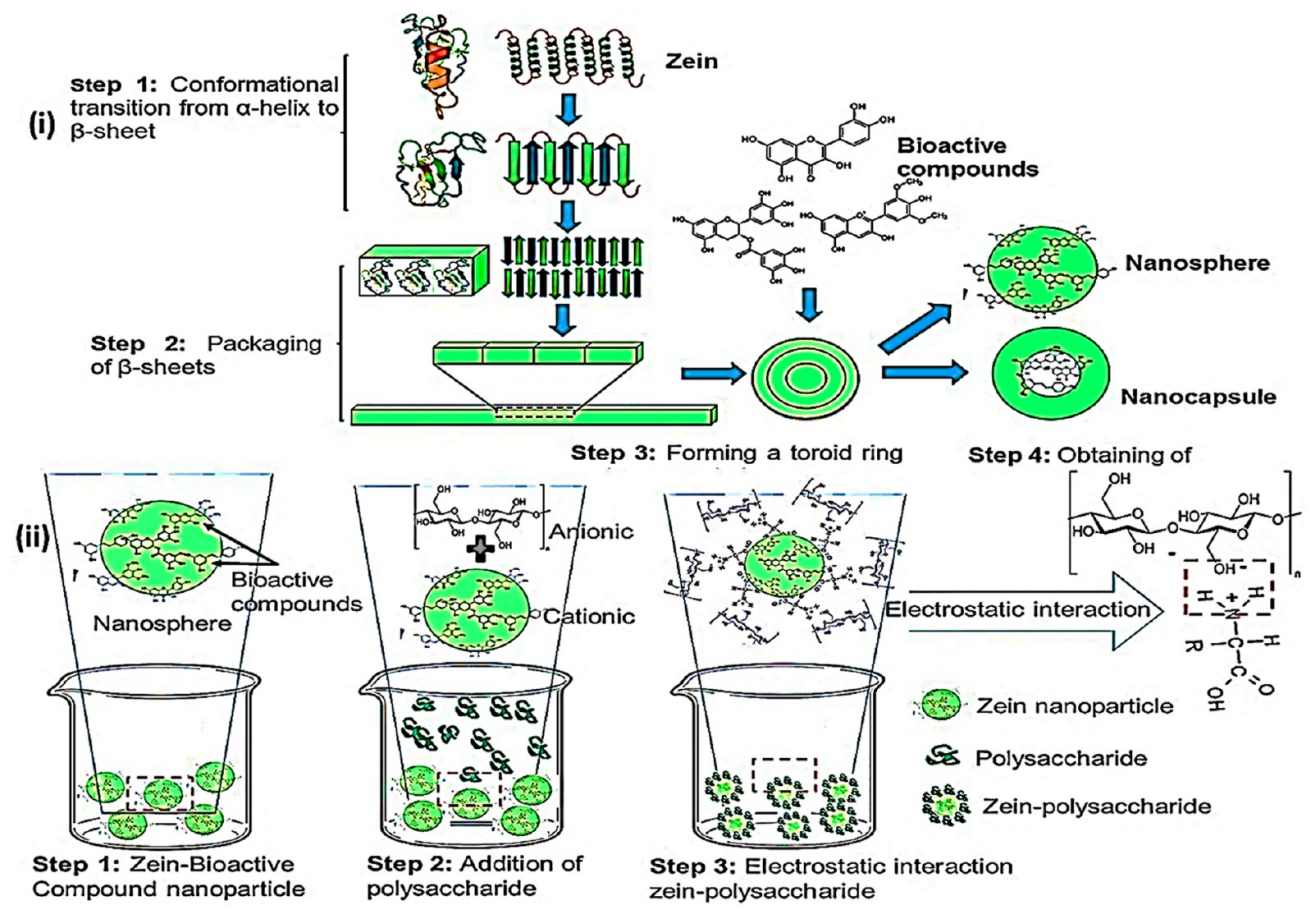
Synthesis and mechanisms of the mode of action of zein NPs. (i) Core–shell compounds and entrapment compounds as the basis for the production and mechanism of zein-based NPs. (ii) Zein-polysaccharide nanoparticles were created using electrostatic electrodeposition to bind antioxidant chemicals. Reproduced from ref (32). Copyright 2018 Elsevier.

Zein is a protein derived from maize and belongs to the class of prolamins; it is usually manufactured from corn gluten meal as a powder and is soluble in aqueous alcohol and used for the preparation of nanoparticles for food, medical, and various other applications.^66−68^ Zein possesses the property of encapsulating different compounds to provide stability and control their release. For this purpose, electrospraying, electrodynamic atomization, and associative phase separation have been considered. The most conventional approach is the preparation of zein by dissolving in ethanol, followed by the precipitation of proteins by adjusting the pH (Figure 2).^69−72^ A helical structure of zein was proposed where the hydrogen bonds stabilized the antiparallel arrangement of nine homologous repeating units resulting in a slightly asymmetric protein molecule.^64,73,74^

Zein possesses a spherical structure with conformational properties similar to conventional globular proteins like ribonuclease and insulin.^75^

**Figure 3:**
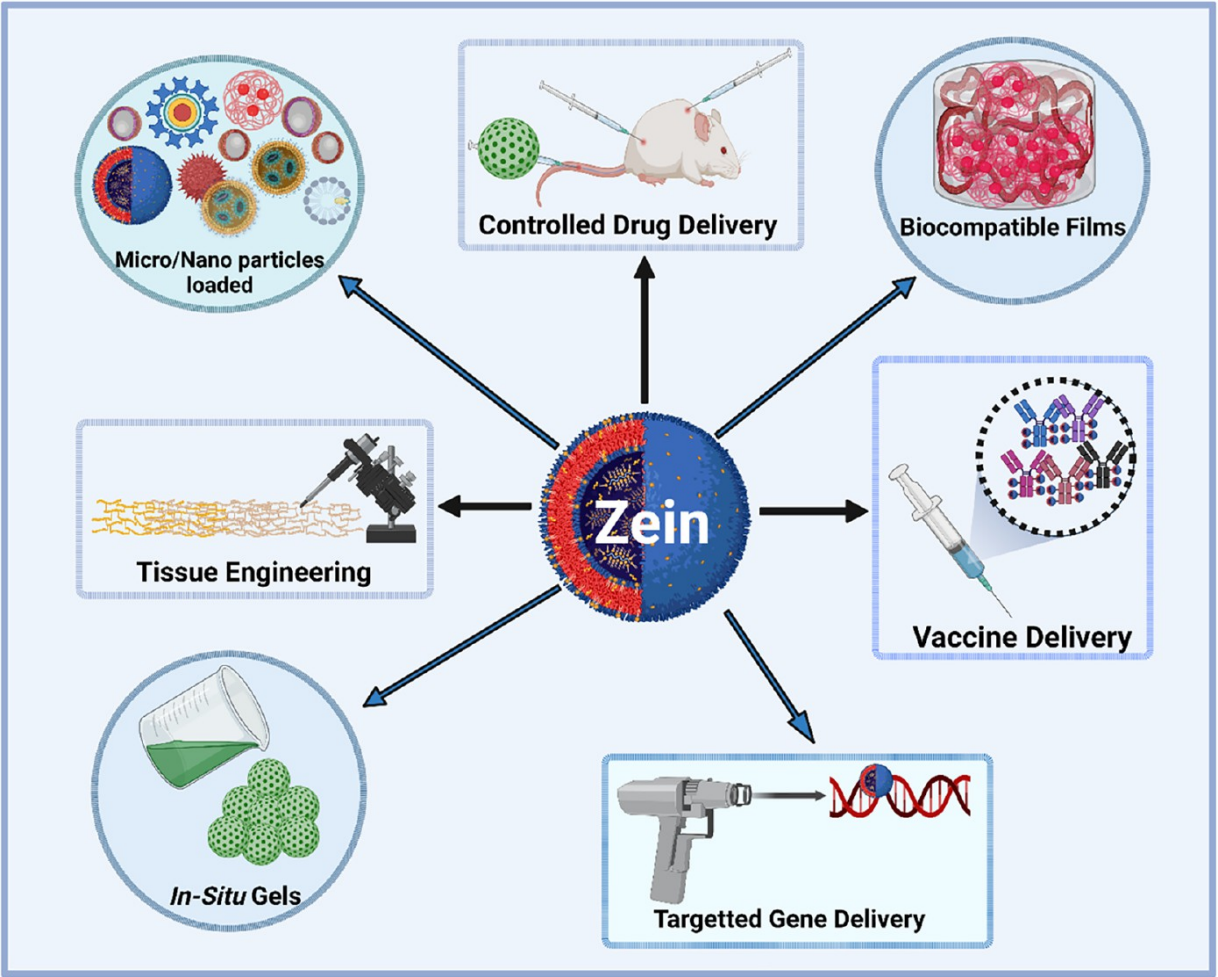
Projection of zein-NP-assisted challenges posed in the clinical setting for targeted drug delivery in precision medicine.

Zein nanoparticles (NPs) demonstrate specific characteristics including particle size, polydispersity index (PI), and encapsulation efficiency. Particle size and zeta potential are typically determined using photon correlation spectroscopy (Figure 3). Despite occasional reductions in value, zein shows considerable potential in various fields such as specialty food, pharmaceuticals, and biodegradable plastics. Since the early 1900s, several experiments have been undertaken on various zein production techniques, although few seem to have had significant economic success. In the 1990s, the pace of research in this field picked up once more, with most of the effort going on to lower the number of solvents needed, extract the solvent, and reuse it cheaply. Future commercial endeavors are likely to increase as the uniqueness of zein as an industrial and specialty polymer is further recognized. The structural architecture of zein has unique solubility criteria. The absence of amino acid residues such as lysine, tryptophan, histidine, and arginine makes zein an unusual protein from all other proteins.^55^

The temperature and pH play essential roles in maintaining the structure and conformation of zein protein. Plant proteins can be affected by thermal treatment leading to conformational changes.^55,76,77^ Zein was diluted in 70% ethanol and heated three times at three different temperatures (75, 85, and 95 °C) (15, 30, and 45 min). On treating the protein with heat, structural changes were observed along with some physical, thermal, and morphological changes. To evaluate the structural alterations in the zein protein, transmission electron microscopy, light scattering dynamically, and fluorescence spectroscopy with UV spectrophotometer, circular dichroism, and differential calorimetry techniques were used.^55,78^ Thermal treatment at 75 °C for 15 min resulted in a narrow particle size distribution, an increase in α helix, a decrease in β sheets, and an enhanced thermal stability. The same experiment was carried out at 85 °C for 30 min showing the formation of zein aggregates with larger size and random increase of coils and decrease in β-turn along with increased fluorescence intensity with changed morphology of the zein protein.^55^ The experiment conducted at three different temperatures at three additional time intervals showed that a native zein protein, when treated at a low temperature, showed partial protein folding followed by extensive unfolding and finally forming protein aggregates when treated for a long time with both heat and high-pressure homogenization.^55^ The thermal treatment of zein NPs resulted in the dispersion property of the colloidal system and the rearrangement of the protein.^47^ Protein accumulation occurs due to the destruction of the secondary and tertiary structure of the protein caused due to high-temperature treatment of the protein.^79^ Despite the denaturation of the protein, the heat treatment also helps the zein protein to form stable NPs.^36,53,79^ Raman analysis also concluded that thermal treatment induced redistribution of amino acid residues on the surface of zein NPs.^79^

pH is an important factor affecting a protein’s overall structure and morphology, resulting in conformational changes. The net charge of zein is 6.2, enough to create aggregates.^44,80−82^ The impact of acids and bases causes changes in zein’s structural, rheological, and antioxidant properties. Zein ethanol solution 70% was allowed to react at room temperature for 24 h at different pH levels, such as neutral (6.5), two acidic levels (2.7, 3.3), and two basic levels (10.5, 12.5). As a result of this different pH level, the glutamine residue of the alpha helix was affected significantly, causing the deamination at both the acidic and basic pHs, resulting in the emulsifying property of zein.^79,83^ SDS-PAGE analysis of zein protein revealed no alterations in either molecular weight or protein polymerization. However, it was observed that the antioxidant property of both the acidic and basic treated zein was enhanced.^54,84^ The alkaline pH controls the nanoprecipitation of zein protein carriers, thereby studying the stability and characteristics of the protein. A recent study showed that, when encapsulated with curcumin by basic deamination treatment, a recent study showed enhanced solubility, strength, and antioxidant properties.^84,85^

## 3.0 Preparation and Synthesis of Zein Nanocarriers

**Figure 4:**
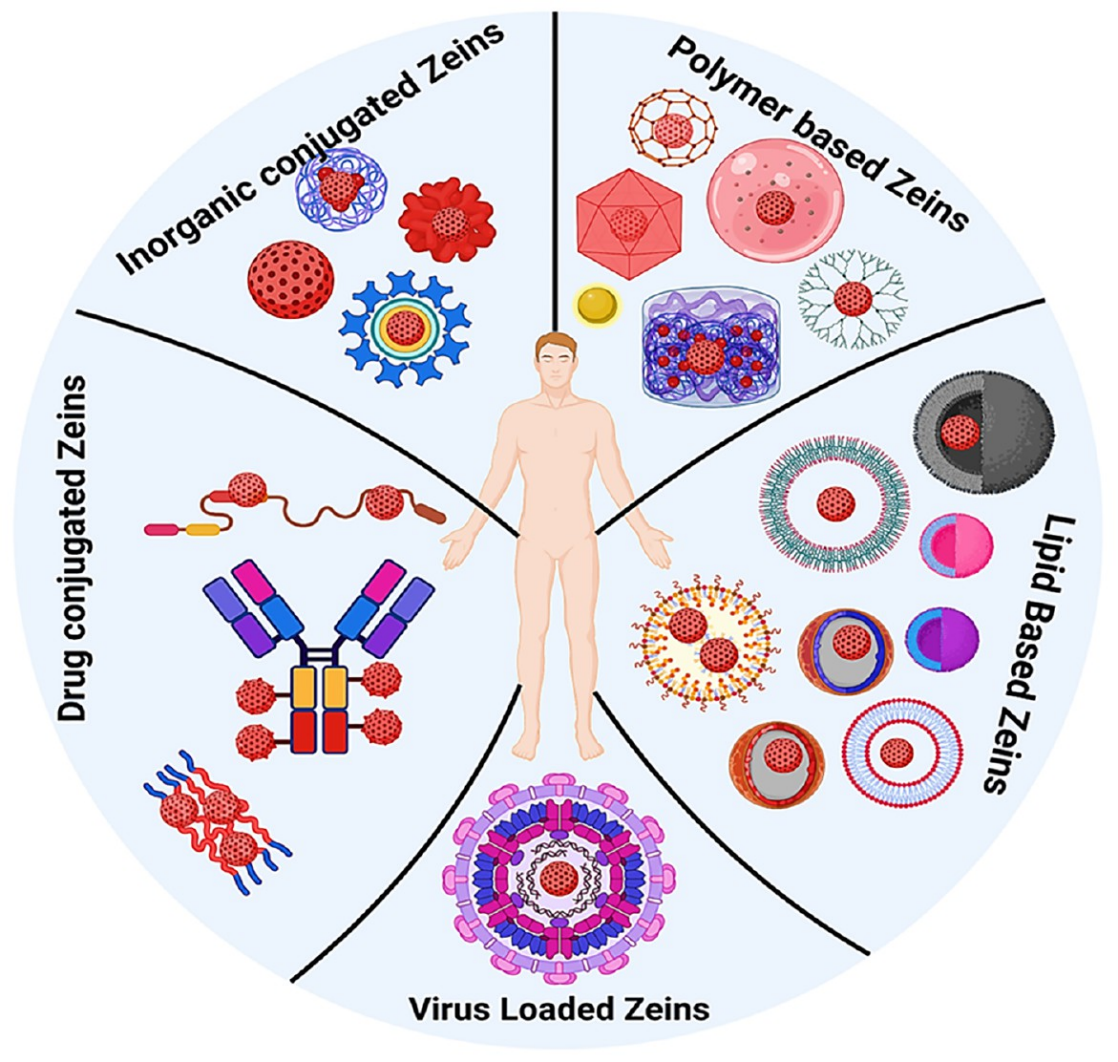
A graphical schematic showing the functionalization and conjugation of zein NPs with the help of different kinds of ligands for desired drug delivery systems and approaches for cancer theranostic applications .

Biodegradable NPs can be obtained from natural as well as synthetic polymers,^75^ but natural polymers, mainly protein-based polymers, are advantageous because of their higher availability and low cost^75,86^ (Figure 4). Zein preparation requires 55–100% alcohol concentration by dispersing zein in ethanol followed by the addition of Tween 80 and PVP^75^ and reports that higher alcohol concentration results in smaller zein NPs.^75,87^ In contrast, pH plays a vital function in the preparation of zein NPs since zein having an isoelectric point of 6.2 is negatively charged when pH is above the isoelectric threshold and positively charged when pH is below the isoelectric point, thereby interfering in the particle size.^50,63,88^ So, further studies are needed to understand the effect of pH on the particle size of zein.

Lecithin and Pluronic F68 are the most stable stabilizers for zein as lecithin can bind well with zein preventing particle aggregation. But the insolubility of lecithin in alcohol often hampers the effective binding of the stabizer to zein. On the other hand, pluronic can be attributed to the formation of stable zein NPs without the aggregate.^89^

**Figure 5:**
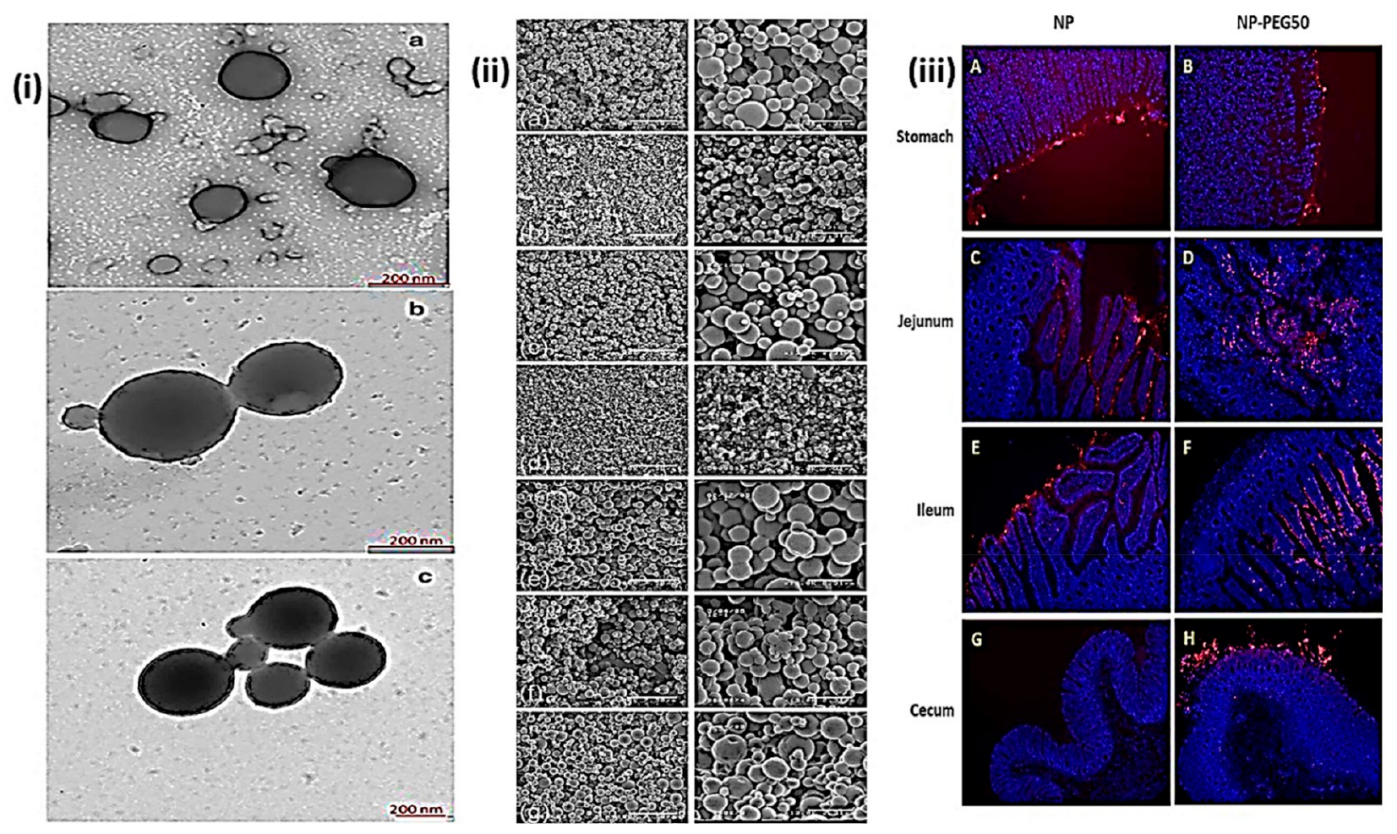
Microscopic images of zein NPs under varied conditions. (i) Electron microscopy tomography of zein-based nanoparticles. (a) Empty uncoated NPs. (b) Insulin-loaded NPs without a coating (I-NP). (c) Insulin-loaded NPs with GT coating (I-GT-NP). The experimental settings were as follows: a 0.1 insulin-to-zein ratio. Reproduced from ref (45). Copyright 2020 Springer. (ii) SEM images of zein NPs made with (a) pure pentane (negative control); (b, c) Sp-85; (d) Sp-80; (e) Tw-80; (f) Bj-92; and (g) OA. The magnification (from left to right) is 4000 and 13,000, and the scale bars (from left to right) are 7.5 and 2.31 m, respectively. Reproduced from ref (89). Copyright 2007 Taylor & Francis Online. (iii) Microscopical fluorescence images of bare NPs and PEG-coated NPs with a PEG-to-zein proportion of 0.5 (NP-PEG50) in animal gastrointestinal tract slices 2 h after delivery. Animal stomach slices are shown in A and B, the jejunum in C and D, the ileum in E and F, and the cecum in G and H. Cell nuclei stained with DAPI are visible in blue. Reproduced with CC-BY license from ref (90). Copyright 2021 Elsevier.

PEG-coated zein NPs with mucous permeating properties facilitate oral drug delivery and other clinical developments (Figure 5). Zein NPs were prepared by desolvation and then coated with PEG, which was detected by electron microscopy and corroborated using FTIR.^90^ The PEG-coated nanoparticles were more durable than other coating materials since PEG had a low polydispersity index and further analysis with the help of SEM and TEM has shown homogeneous populations of all the nanoparticles and the presence of a slightly less dense substance was achieved. Interestingly, these NPs based on protein possess the ability to encapsulate small hydrophobic molecules and hydrophilic macromolecules.^45^ However, conventional zein NPs did not provide the necessary scope of bioavailability for clinical application; hence these oral nanocarriers were developed without any new chemical entities in their formulation with mucus permeating properties.^91^ By using a solution-enhanced dispersion by the supercritical CO_2_ (SEDS) technique, zein can be employed as a carrier for delivering active components to develop controlled release medications, where the nozzle structure and CO_2_ flow rate significantly affect the morphology and size of the particles as well as its distribution on the velocity field and impacted by computational fluid dynamics.^92^ Raw and processed zein samples were screened through XRD patterns to obtain a diffraction angle,^82^ implying an increase in intensity^48^ and studying the amorphous structure of zein NPs successfully through the SEDS approach having significant impacts on nozzle structure and SC-CO_2_ followed by CFD simulations.^45^

**Figure 6:**
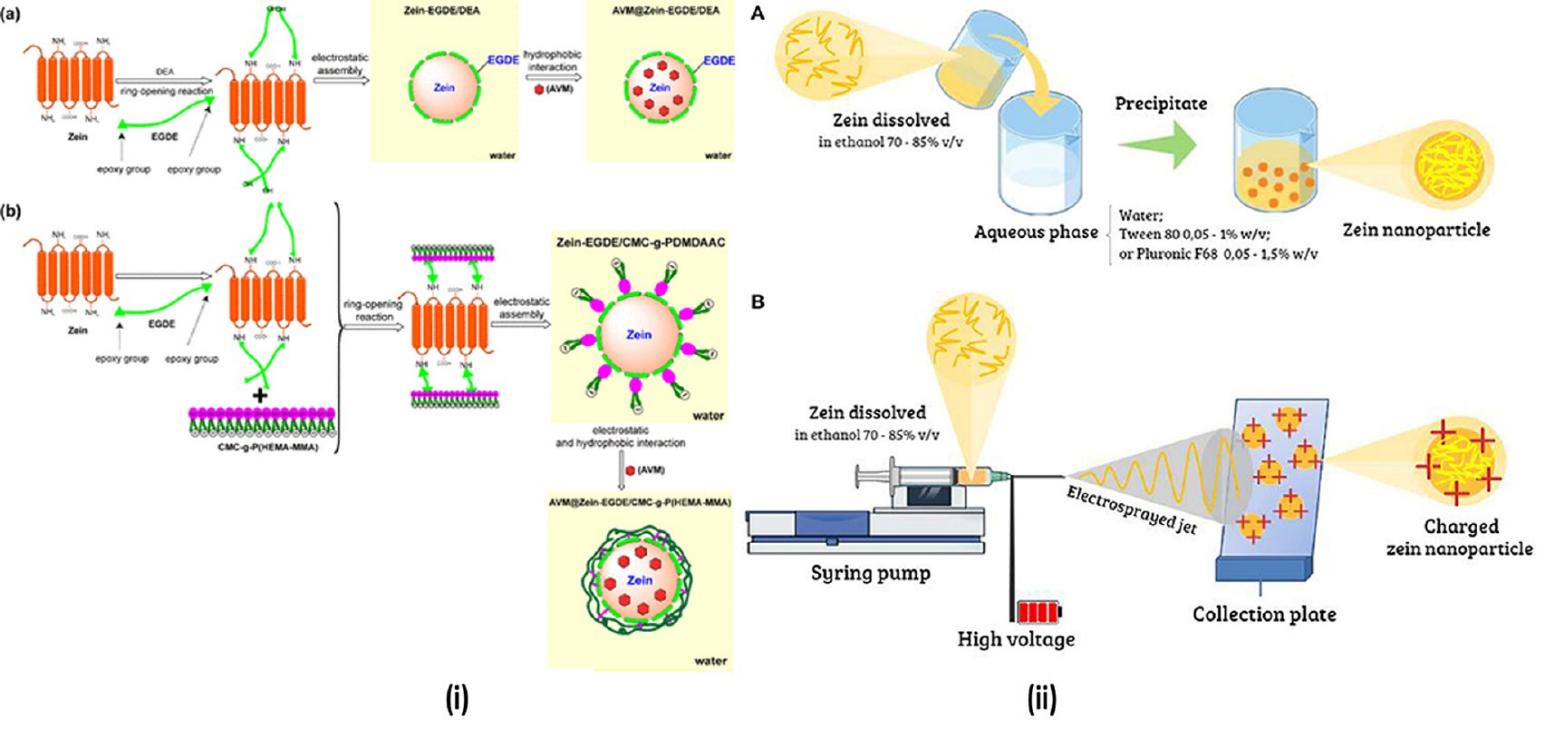
Synthesis and characterization of zein nanosystems for anticancer therapy. (i) Diagrams of AVM contained in both zein-EGDE/DEA and zein-EGDE/CMC-*g*-P. (HEMA-MMA).^60^ Reproduced from ref (60). Copyright 2020 ACS. (ii) Methodologies for preparing zein NPs vary: (A) strategies for antisolvent precipitation/liquid–liquid dispersion/phase separation and (B) zein electrohydrodynamic atomization nanocarrier for producing charged zein NPs.^81^ Reproduced with CC-BY license from ref (81). Copyright 2018 Frontiers.

Zein extraction can be achieved by an ample method, which uses single-column extraction via stepwise distillers dried grains with solubles (DDGS) (Figure 6). The initiation is attained using a single packed column for oil extraction from the DDGS with an extended purpose of adding DDGS and *n*-hexane to the column, representing the extraction solvent. The column is subjected to heat, and the operating conditions are set per the requirement, followed by residue collection from the oil extraction and regulated operating conditions. This step progressed after encountering three extraction cycles, allowing the solvent to evaporate, followed by residue washing with distilled water. Zein yield post centrifugation and drying step is approximately 30.7%.^34^ Another means of extraction is performed postanalysis with isobutanol and ethanol. A requisite amount of DDGS is added to the chosen solvent and anhydrous sodium sulfate under optimum conditions, while constant stirring is done for the homogenization of the solution. The obtained product is filtered and cooled down to 4 °C, followed by the addition of distilled water and allowing storage at the same temperature. The final step is centrifuging the solution separating the extract into soaked zein and drying the section at 40 °C.^34,64,75^ The role of peptide NPs in the interactions of antioxidants in Pickering emulsions is regulated by using a kinetic model to analyze the distribution of gallic acid (GA) in zein NP emulsions (ZPEs). GA bonded to zein NPs via hydrogen bonding manifested enhanced levels of GA upon elevation of the zein NP concentration, thereby establishing a direct correlation between oxidative stability and zein NP concentration. The tailored zein particles were utilized as interfacial stabilizers of emulsion droplets owing to the higher absorption of zein NPs, further upregulating the interfacial concentration of GA.^51,93−95^ The percentage GA modulated by the interfacial loading content of zein NPs on the oxidative stability despite the physical filter effect plays a considerable role in antioxidation effects. Hence, the distribution of phenolic antioxidants is linked with the emulsion systems along with other factors such as the charge and size of the NPs, and extensive research digging into the designing of these interfaces in emulsions can lead to better effective and rational systems.^96^

## 4.0 Gene Therapy by Zein Nanoparticles for Cancer Therapy

Zein is a protein NP that possesses unique merits that include minimal toxicity, drug binding capacity, and enormous renewable sources that allow zein-encapsulated NPs to effectively and efficiently be used in different types of diagnosis, including cancer therapy.^97−99^

Nanocarriers have a high ratio of surface area to volume that can improve the biodistribution and the pharmacokinetics of zein bound drug as a therapeutic agent with high permeability that makes it ideal for gene delivery and generally regarded as safe (GRAS) as a carrier in drug delivery.^51,56,100^ Gene therapy is a very recent approach in the treatment of various cancers, one of them being hepatocellular carcinoma, which usually presents at an advanced stage with a poor prognosis. The detection of hepatocellular carcinoma at an early stage is still being researched; however, it was found that gene therapy could be a hope of light though the success rate dramatically depends on the development of the vector and how effectively the gene is delivered to the target cell.^58,101,102^

PTEN (phosphatase tensin homologue) and TRAIL (tumor necrosis factor related apoptosis-inducing ligand) loaded zein NPs are used as an effective therapy for hepatocellular carcinoma. PTEN, a tumor suppressor gene, can inhibit proliferation, migration, and invasion of HepG2 liver cell lines and promote cell survival and growth through its aggressive and phosphatase action.^103^ Specific molecules inhibiting TRAIL-mediated cell death cause HepG2 cells resistant to TRAIL, thus showing an antiproliferative effect.^103^ As a result of PTEN and TRAIL-loaded zinc nanoparticles, markers of apoptosis (p53), angiogenesis (VEGF), and metastasis (MMP-2) in animal liver tissue were expressed at the mRNA level. The increased expression of p53, an ideal target for restoring the apoptotic pathway and silencing carcinogenesis was observed.^103^ It can hamper cell growth by interfering with cell cycle processes and causing cell apoptosis.^103^ However, luteolin possessing poor aqueous solubility is limited by its oral bioavailability; hence, improvement is made using zein protein and sodium caseinate that acts as a nanocarrier. When encapsulated with zein, the antioxidant activity changes with luteolin, enhancing cytotoxicity against colon cancer and inducing apoptosis. It is reported that NPs for cancer therapy are usually between 70 and 280 nm and can effectively target vital organs of the body.^103^

The luteolin-encapsulated zein NPs act as a therapy for colon cancer and are based on the Hixson–Crowell model that is widely accepted for the oral delivery of drugs. Exemestane (EXM) and resveratrol (RES) are used as medications for breast cancer. Still, due to their poor solubility and low permeability, these are encapsulated using zein, thereby enhancing the cytotoxicity of breast cancer cells.^51^ In conclusion, nanocarriers could be an exceptional approach to eradicating cancer by therapy and offer a potential advancement in the treatment strategy.

## 5.0 Zein Nanosystems for Drug Delivery in Anticancer Therapy

Classical chemotherapeutic drugs have found only limited clinical use in the fight against cancer because of their low solubility, lack of selectivity, and undesirable side effects. Presently, cancer-targeted nanotechnology has led to the creation of novel materials with different functions for delivering drugs, describing the strategy, and bioimaging to overcome these constraints.

Zein protein’s unusual thermodynamic features have led to several applications in diverse industries, including food and healthcare. Notably, in recent years, there has been a lot of interest in developing zein-based delivery trucks (Figure 6). In this regard, zein, a proline-rich protein, has shown the capacity to entrap and protect active molecules with a more significant proportion of hydrophobic amino acid, demonstrating the ability to extend the plasma level of the drug and thus overcome the disadvantage of hydrophilic polymer to achieve sustained release.^65,105^ The structural features of zein make it capable of protecting loaded compounds from harsh stomach conditions and offer a mechanism for controlled release.^57^

**Figure 7:**
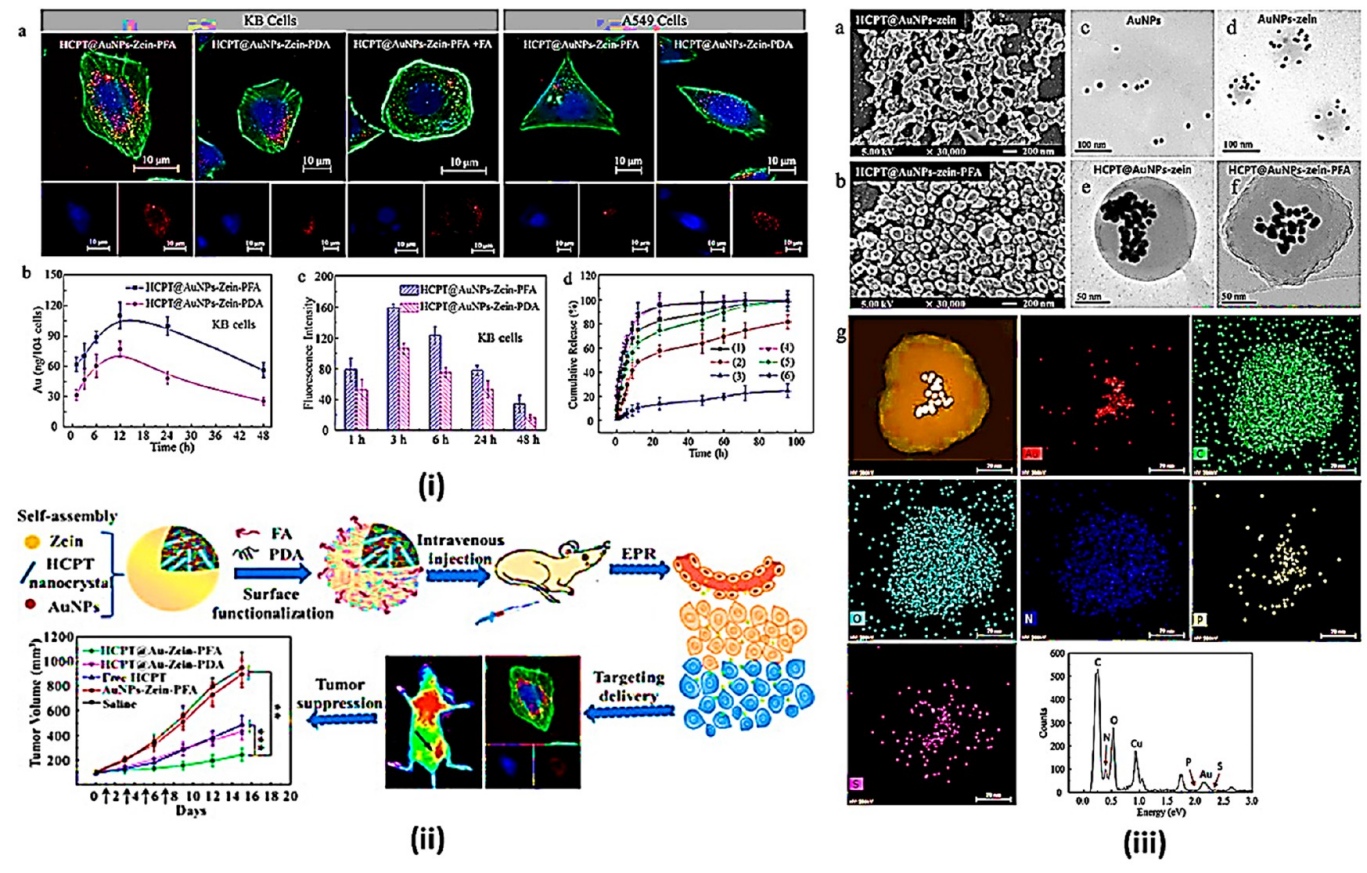
Projection of encapsulation of zein NPs for targeted drug delivery and gene therapy. (i) Cancer cells take up and release HCPT-loaded NCs. (a) CLSM images of KB and A549 cells treated for 1 h at 37 °C with HCPT-loaded NCs (or HCPT@AuNPs-zein-PFA NCs in the presence of an excess of free folate). Alexa Fluor 488 phalloidin was used to stain the plasma membranes of cells in green. Their nuclei were dyed blue by DAPI. The NPs were labeled in red with cy5. (b) AuNP concentration in cells and (c) KB cell fluorescence intensity after different time intervals of exposure to HCPT-loaded NCs. (d) At 37 °C, HCPT was released *in vitro* from HCPT-tethered NCs or HCPT crystals in acetate buffer at pH 5.0 or in an enzymatic environment. (1) At an enzymatic environment, HCPT@AuNPs-zein-FA NCs; (2) pH 5.0 acetate buffer containing HCPT@AuNPs-zein-FA NCs; (3) pH 7.4 HCPT@AuNPs-zein-FA NCs; (4) HCPT crystals at enzymatic environment; (5) HCPT crystals in an acetate buffer with a pH of 5.0; (6) At pH 7.4, HCPT crystals. The data is presented as the mean standard deviation (S.D.) of three separate studies. (ii) A graphical model. (iii) SEM images of HCPT@AuNPs-zein NCs (a) and HCPT@AuNPs-zein-PFA NCs (b). TEM images of (c) AuNPs-zein, (d) AuNPs-zein NCs, (e) HCPT, and (f) HCPT@AuNPs-zein-PFA NCs. The quantitative analysis and elemental EDX mapping of a single HCPT@AuNPs-zein-PFA NP (g). Reproduced from ref (104). Copyright 2017 Elsevier.

Further zein NP formulations are being researched in cancer due to the hydrophobic surface and low net charge at pH over 5 (since zein is near its isoelectric point 6.2). Due to its inherent biocompatibility, nontoxicity, *in vivo* biodegradability, and capacity for self-assembly because of the plant-derived agents, zein could be a successful method in this system. Precision drug delivery has been a problem in the tumor microenvironment for quite some time, and the application of zein NPs for targeted drug delivery could mitigate the problem (Figure 7).^106−108^ Herein we have discussed the clinical significance of zein NPs for drug delivery of anticancer compounds, summarized in Table 1.

**Table 1:** Clinical Significance of the Application of Zein NPs for Anti-Cancer Therapy

Nanoparticles	Model of Preclinical	Drug Delivery	Functional Ligand	Mechanisms	Reference
Zein nanoparticles	*In vitro*	Metal tannic acid	N/A	Spherical configuration and strong encapsulation. Reducing and stabilizing agents.	(109)
Zein nanoparticles	N/A	Doxorubicin	Pectin hydrogels	Slow release of drugs, increased storability, and a suitable environment for drugs.	(110)
Zein nanoparticles	*In vitro*/*In vivo*	Docetaxel	Chondroitin sulfate	Shows zero cytotoxicity and has pharmacokinetic behavior during delivery of drugs.	(111)
Zein nanoparticles	*In vitro*	Doxorubicin	Nocodazole and cytochalasin D	Intercalation into DNA and disruption of topoisomerase-II-mediated DNA repair and generation of free radicals and their damage to cellular membranes, DNA and proteins.	(100)
Zein nanoparticles	*In vitro*	AuZNS	Glycol	Photothermal efficacy, nontoxic, and biocompatibility properties are up to the mark.	(112)
Zein nanoparticles	*In vitro*	Rapamycin and wogonin	Lactoferrin	Disrupt cytokine signaling that promotes lymphocyte growth and differentiation.	(59)
Zein nanoparticles	*In vivo*	Vorinostat, bortezomib	N/A	It inhibits HDAC1, HDAC2, HDAC3, and HDAC6 at nanomolar concentrations.	(113)
Zein nanoparticles	*In vitro*	Sodium deoxycholate	Paclitaxel	These showed good efficiency in maintaining the pH and also maintained the PH at 50° which will not affect the structure, showing good entrapment with high efficiency.	(87)
Zein nanoparticles	*In vitro*	Maytansine	N/A	Splenic tissue of DM1 shows reduced white pulp and lymph, resulting in spleen damage.	(1)
Zein nanoparticles	*In vitro*	Paclitaxel	N/A	Paclitaxel targets microtubules. At high concentration, PTX causes mitotic arrest at the G2/M phase, whereas, at low concentration, apoptosis is induced at the G0 and G1/S phase either via Raf-1 kinase activation or p53/p21 depending on the dose concentration.	(61)
Zein nanoparticles	*In vitro*	Beta-carotene	N/A	Entrapment efficiency as well as micrometric behavior, also shows results in MCF-7 cells.	(115)
Zein nanoparticles	*In vitro*	Resveratrol	N/A	Resveratrol exhibits anti-inflammatory effects and immunomodulating functions via sirtuin-1 (Sirt-1) activation.	(120)
Zein nanoparticles	*In vitro*	Lovastatin	N/A	Entrapment efficiency against cells and particular particle size and good zeta potential and it is also susceptible to exhibiting antiproliferative behavior against HepG2 cells.	(116)
Zein nanoparticles	*In vitro*	Curcumin	Hyaluronic acid	Cyto-suitability as well as hemo-adaptability.	(117)
Zein nanoparticles	*In vitro*	Paclitaxel	PEGylated	PTX binds to microtubules instead of tubulin dimers and stabilizes microtubules (polymerization) by promoting the assembly of alpha and beta tubulin subunits, the building blocks of microtubules.	(114)
Zein nanoparticles	N/A	Phosphatidylcholine	Indocyanine	Photo-cytotoxicity, indocyanine green by embedding it in liposomes.	(118)
Zein nanoparticles	*In vitro*	Paclitaxel and sodium deoxycholate	N/A	Drug entrapment efficiency is increased due to suitable storage stability, and not destabilized by temperatures of up to 50 °C.	(87)

**Figure 8:**
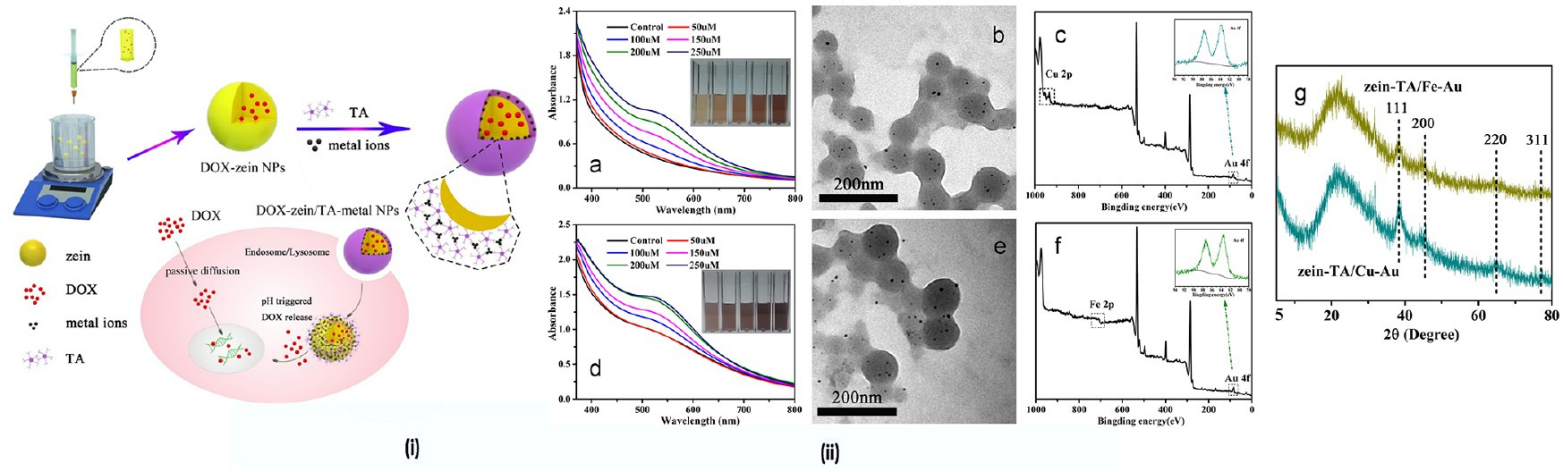
Zein NPs loaded DOX for anticancer therapy. (i) A graphical model explaining the loading of DOX with zein NPs for anticancer therapy. (ii) UV–vis absorption spectra of Au@zein-tannic acid (TA)/Cu^II^ NPs (a) and Au@zein-TA/Fe^III^ NPs (d) with different Au concentrations. TEM images of Au@zein-TA/Cu^II^ NPs (b) and Au@zein-TA/Fe^III^ NPs (e) at a Au concentration of 200 μM. XPS survey spectra of Au@zein-TA/Cu^II^ NPs (c) and Au@zein-TA/Fe^III^ NPs (f) at a Au concentration of 200 μM. XRD patterns of Au@zein-TA/Fe^III^ NPs and Au@zein-TA/Cu^II^ NPs (g) at an Au concentration of 200 μM. Reproduced from ref (109). Copyright 2015 Elsevier.

**Figure 9:**
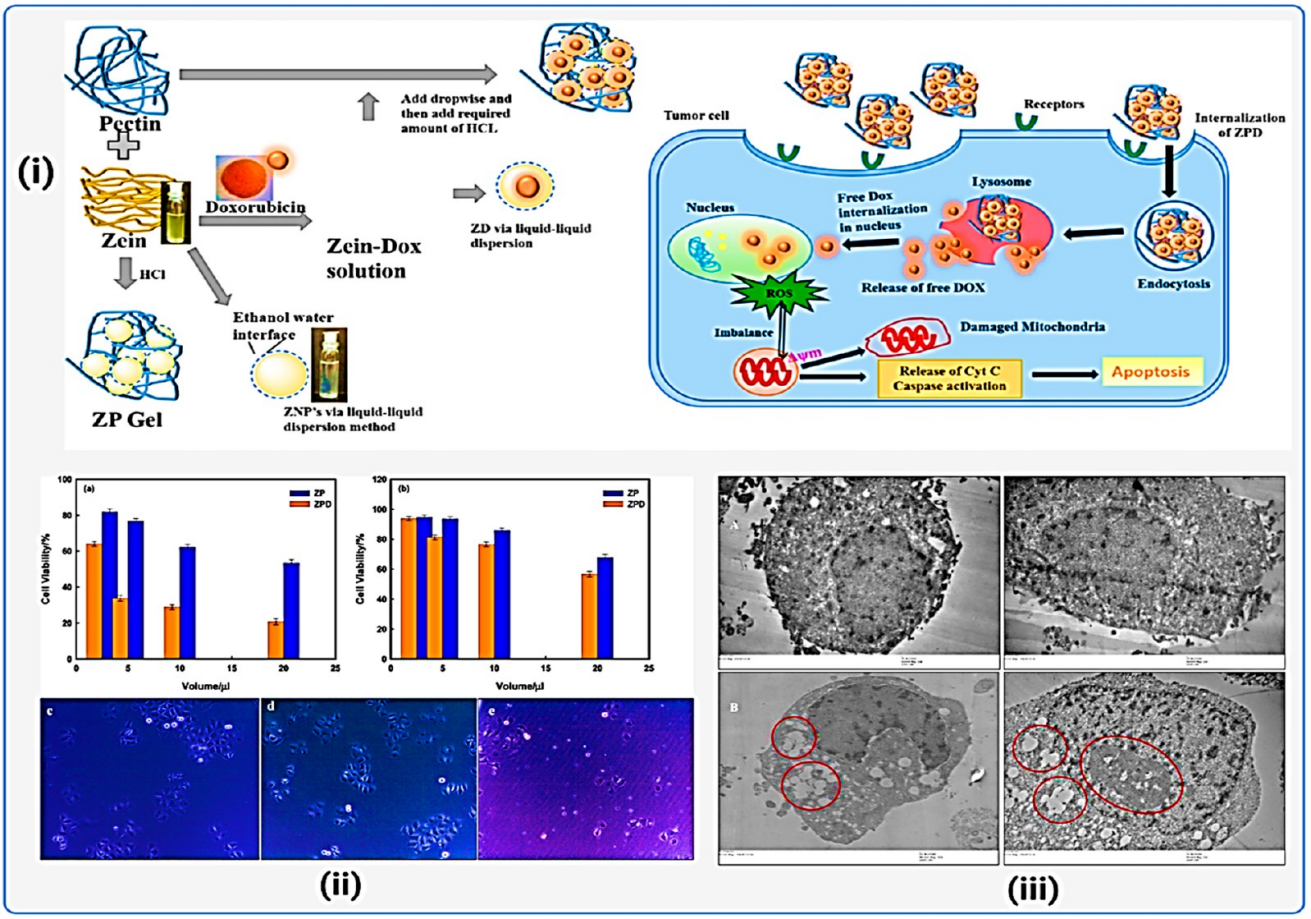
Formation of zein pectin doxorubicin (Dox) hydrogels for precision drug delivery in HeLa cells. (i) Graphical model. (ii) Hydrogel cytotoxicity: The impact of ZP and ZPD hydrogels on the survival of (a) Hela and (b) HEK293 cell lines via an MTT assay. Cell morphology analysis: (c) cell control, (d, e) treated with ZPD hydrogels. (iii) EM micrographs of (A) untreated and (B) treated cells showing considerable ultrastructural alterations documented at several magnifications. Reproduced from ref (110). Copyright 2020 Elsevier.

Liang et al. used an *in vitro* liver cell line with tannic metal acid enclosed with zein NP for the delivery of the anticancer drug DOX for PH response (Figure 8). The disintegration of the metal TA film was modulated by altering the metal category and the value of PH.DOX/zein-TA/Cu (III) NP, which measured 178.9 nm, and DOX/zein-TA/Fe (III), which measured 188.5 nm. The former exhibited a spherical configuration, while the latter demonstrated strong encapsulation efficiency. Au NPs were made through zein-TA/metal NPs which will act as reducing and stabilizing agents. Thus, Au@zein-TA/metal NPs were used in cancer radiation treatment because of a considerable amount of surface-plasmon-resonance (SPR)-enhanced uptake.^109^ In a similar study, Kaushik et al. used doxorubicin-loaded zein NPs joined with pectin hydrogels in hela drug-doxorubicin cells for anticancerous scaffolds (Figure 9). The targeted drug delivery exhibited reduced cell viability, increased reactive oxygen species (ROS) production of pectin hydrogels, and changes in the cell shape. The conjugation of hydrogel preparation with zein NPs results in the slow release of drugs, increased storability, and a suitable environment for drugs like temperature, pH, enzyme, light, and bioreducible environment. Hence, inorganic hydrogels are preferable for the treatment of anticancer because of their fewer side effects due to the advantages of their biocompatibility, retention of drug potency by increasing half-life, and controlled release with minimal collateral damage to neighboring tissues.^110^ A similar investigation was conducted by Dong et al., who used *in vitro* biodegradable self-assembly zein NPs with doxorubicin to treat cancer. Doxorubicin (DOX), composed of zein NPs, exhibits 200 nm, spherical with an acidic pH (5.0–6.5) and a long-term drug release. DOX-zein-NPs have an antiproliferative effect on HeLa cells, and DOX-zein NPs kill more HeLa cells than free DOX. Zein structure can manage the delivery of DOX wherein DOX-zein-NPs were found in HeLa cells. Nocodazole and cytochalasin D suppressed nanozein-DOX endocytosis, which indicates that macropinocytosis is the endocytosis pathway of DOX-zein-NPs. Hence zein nanoencapsulation is used to treat cervical cancer.^100^

**Figure 10:**
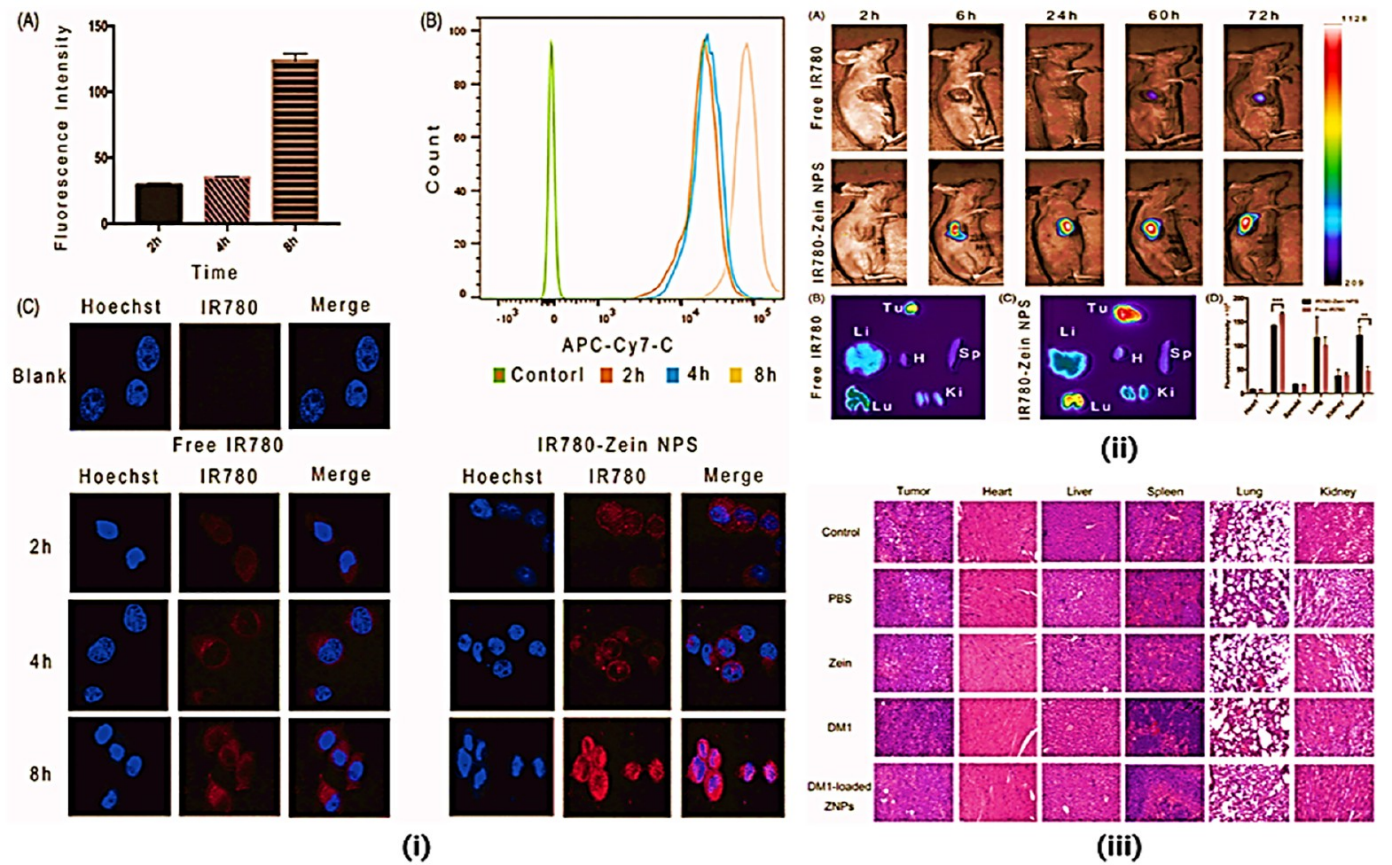
Zein NP mediated drug delivery for lung cancer regression. (i) (A and B) Flow cytometry data of A549 cells incubated with IR-780-loaded ZNPs by recording IR-780 fluorescence. (C) Confocal fluorescence images of A549 cells incubated with IR-780-loaded ZNPs for 2, 4, and 8 h relative to control untreated cells. Blue and red colors represented Hoechst-stained cell nuclei and IR-780 fluorescence, respectively. Error bars show SD. **p < 0.01 (*n* = 3). (ii) *In vivo* and *ex vivo* fluorescence imaging. (A) *In vivo* fluorescence images of A549-tumor-bearing nude mice taken at different time points after iv injection of free IR-780 (20 μg) and IR-780-loaded ZNPs (20 μg of IR780 equiv). *Ex vivo* fluorescence images of major organs and tumors dissected from mice injected with free IR-780 (B) and IR-780-loaded ZNPs (C) at 60 h. Tu, H, Li, Sp, Lu, and Ki stand for tumor, heart, liver, spleen, lung, and kidney, respectively. (D) Semiquantitative relative biodistribution of free IR-780 and IR-780-loaded ZNPs in various organs as determined by the fluorescence intensities measured software. **p < 0.01, ***p < 0.001 (*n* = 3). (iii) H&E staining of major mice organs from different groups. The spleen tissue of DM1 indicates the obvious reduction of splenic white pulp and lymph, leading to spleen damage. Many extramedullary hematopoietic cells (red arrows) are in the red pulp, and multinuclear giant cell proliferation (black arrows) suggests inflammation in the spleen. The images were acquired using a Pannoramic MIDI (3DHISTECH, EU) at 20× objective. Reproduced with CC-BY license from ref (1). Copyright 2020 Taylor & Francis Online.

In the case of cellular uptake and drug-loaded strength, Han et al. used zein NPs blended drug-docetaxel with chondroitin sulfate (CS) for delivery of docetaxel for the treatment of prostate cancer that expresses CD44. It constitutes 157.8 ± 3.6 nm of diameter and 64.2 ± 1.9% of encapsulation efficiency of docetaxel. An *in vitro* result depicts the colloidal steadiness and efficiency of cellular uptake of zein NP and zein/CS NP with nearly zero cytotoxicity. *In vivo* results from tumor xenograft mice shows pharmacokinetic behavior for drug delivery in the tumor through the terminal t1/2 and reduced CL (Figure 10).^111^ In addition, Chauhan et al. used the synthesis of gold-deposited zein nanoshells to treat cancer. By depositing a thin layer of gold over glycol, Au conjugated zein nanoshells (AuZNS) were fashioned, containing properties of liposomes and polymer of the same size, i.e., 100 nm, and photothermal efficacy, i.e., 808 nm. They exhibit nontoxic properties, and their biocompatibility properties depict similar results for photothermal therapy for both breast and cervical cancer. AuZNS can increase the temperature from 37 to 43 °C within 1 min via *in vitro*. Due to being environment friendly and having a great future perspective, AuZNS are used for the clinical purpose of cancer therapy.^112^

A similar investigation by Sabra et al. used micelles constituted of amphiphilic zein-lactoferrin for rapamycin and wogonin delivery to breast cancer tumors. Physical stability was observed in both un-cross-linked and GLA-cross-linked micelles. It shows no significant change in their zeta potential and size within 30 days of the period and exhibits a limited hemolytic rate, showing stable results in *in vitro* serum.^59^ The *in vitro* cytotoxicity of GLA-cross-linked micelles against MCF-7 breast cancer cells was improved with cross-linked micelles for an antitumor effect, but there was no such result in un-cross-linked micelles; the development of this effect is major tumor angiogenesis. To overcome this problem combination of new micelles is used, which will reduce the levels of p-AKT and MAPK expression. Thus, zein-lactoferrin is used for the treatment of malignant tissues.^59^

We focus on Thapa et al., the research that used *in vivo* vorinostat, bortezomib, and combination-loaded zein NPs to treat metastatic prostate cancers. An approximately 160 nm size of the particle and approximately 0.20 polydispersity index of ZNP/VB are made. It mainly shows characteristics of more significant cytotoxicity; apoptotic behavior also shows more excellent absorption in various cancer cells in the prostate and can increase the antimigration behavior and pro-apoptotic protein induction.^113^

**Figure 11:**
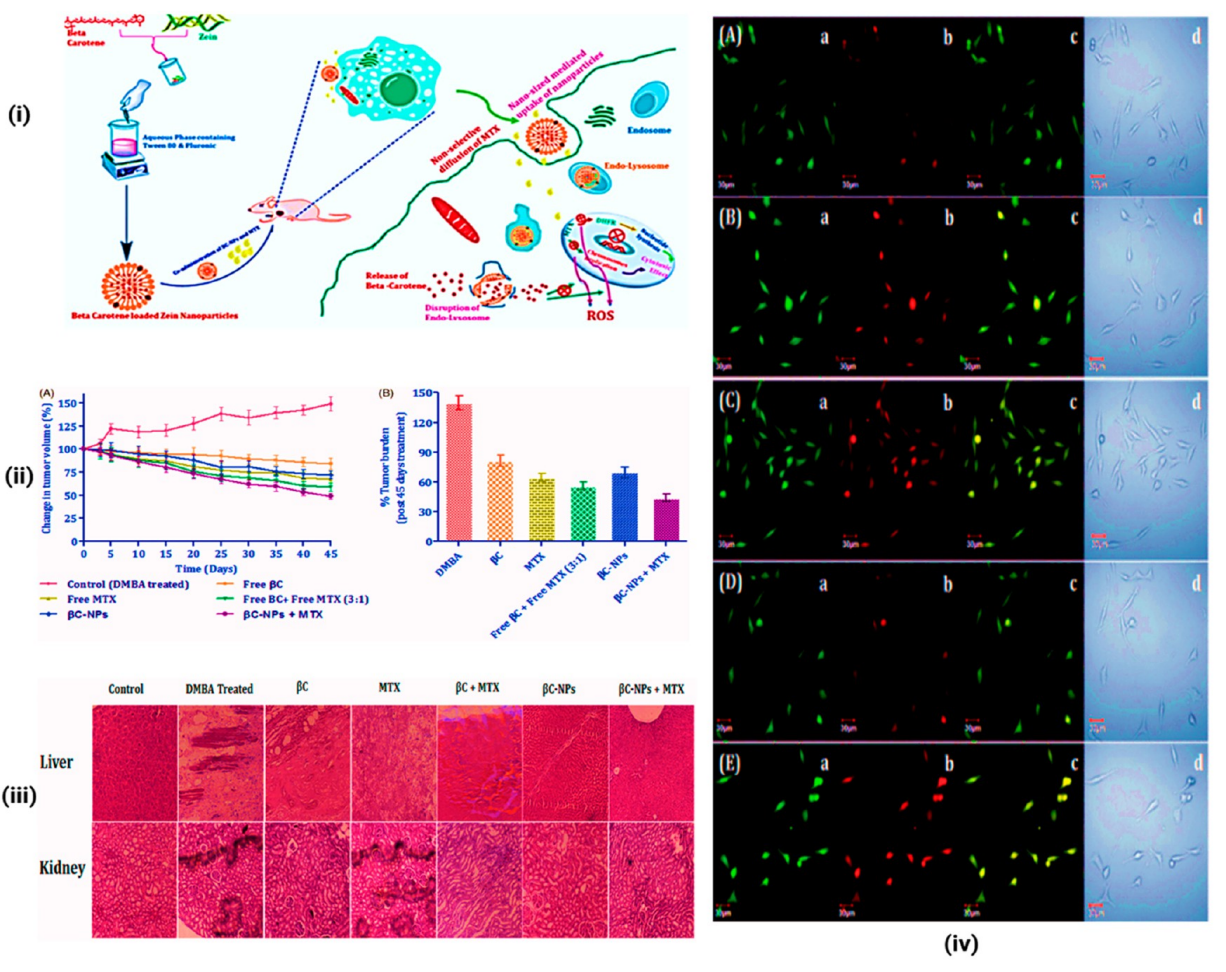
Zein NPs assisted improved cellular uptake and cytotoxicity and exhibited enhanced oral biopharmaceutical performance of beta-carotene (βC). (i) A schematic model combination regimen could also be a promising platform to facilitate the therapeutic benefits of anticancer agents. (ii) Antitumor activity of all tested formulations. (A) Tumor progression after repetitive oral administration of free βC, free MTX, βC+MTX (3:1), βC-NPs+MTX, and BC-NPs+MTX. (B) Assessment of *in vivo* therapeutic activity of βC-NPs in a DMBA-induced breast cancer animal model. Percent tumor burden was calculated after the completion of 45 days of therapy. (iii) Apoptosis assay of different formulations against MCF-7 cells. (a) The green channel reveals the fluorescence from carboxyfluorescein (cell viability marker dye); (b) the red channel depicts fluorescence from the Annexin Cy3.18 conjugate (cell apoptosis marker dye); (c) the third channel shows the overlay image; (d) the fourth window illustrates the differential contrast image of representative cells. (iv) Histological examination of liver and kidney tissues after treatment with control, positive control (DMBA-treated), βC, MTX, βC+MTX (3:1), βC-NPs, and βC-NPs+MTX. Reproduced with CC-BY license from ref (115). Copyright 2018 Taylor & Francis Online.

In another study, Gagliardi et al. used *in vitro* sodium deoxycholate packed paclitaxel with zein NP to treat anticancer. It possesses good efficiency in maintaining the pH in both a temperature-dependent and non-dependent manner, which will not affect the structure of the zein particles. Moreover, they depict enhanced characteristics of good entrapment with high efficiency. But PTX has some flaws: cytotoxicity that is not specific, poor water solubility, and moderate bioavailability.^87^ Hou et al. used a mixer of paclitaxel (PTX), zein, a disulfide linker, and NPs to form a prodrug for targeted delivery of cancer. *In vitro* studies exhibit cancer regression when the combination mentioned above was used for cancer regression with negligible toxicity.^61^ Soe et al. used paclitaxel enclosed with PEGylated zein NPs for cancer treatment. Paclitaxel (PTX)/zein-FA NPs possess ∼180 nm diameter and approximately ∼0.22 polydispersity index (Figure 11). An *in vitro* result shows that PTX causes cytotoxicity, which is further involved by Paclitaxel PTX/zein-FA NPs in KB cells, by stimulating pro-apoptotic proteins and by blocking the antiapoptotic proteins. It also shows antimigratory activity and could change the KB cell cycle profile, while A549 folate receptor-negative cancer cells were not significantly enhanced. *In vivo* results give less toxicity by PTX/zein-FA NPs. Hence PTX/zein-FA NPs are used to treat cancer cells.^114^

Jain et al. used beta-carotene enclosed with zein NPs to treat breast cancer through the phase separation technique, which shows entrapment efficiency and micrometric behavior (Figure 11). Based on the study, it was inferred that cellular absorption, cytotoxicity, and oral biopharmaceutical efficacy of beta-carotene were all improved by zein NPs without toxicity.^115^

On the other hand, Alhakamy et al. prepared Lovastatin mixed zein NP (LVS-ZN NPs) drugs to treat HepG2 cells that accelerate apoptosis. LVS-ZN NPs exhibit excellent features, such as having the best entrapment efficiency against cells, a particular particle size, and good zeta potential. It is also susceptible to exhibiting antiproliferative behavior against HepG2 cells.^100^ The antiproliferative activity of ZN in itself was significantly higher than that of LVS; it also caused significant cell accumulation in the G2/M and pre-G phases shown by cell cycle results exhibiting the best potency of LVS-ZN NPs. The increased pro-apoptotic activity of the prepared formula was established in the pre-G phase. So, lovastatin-based zein NPs are used to treat cancer.^116^

**Figure 12:**
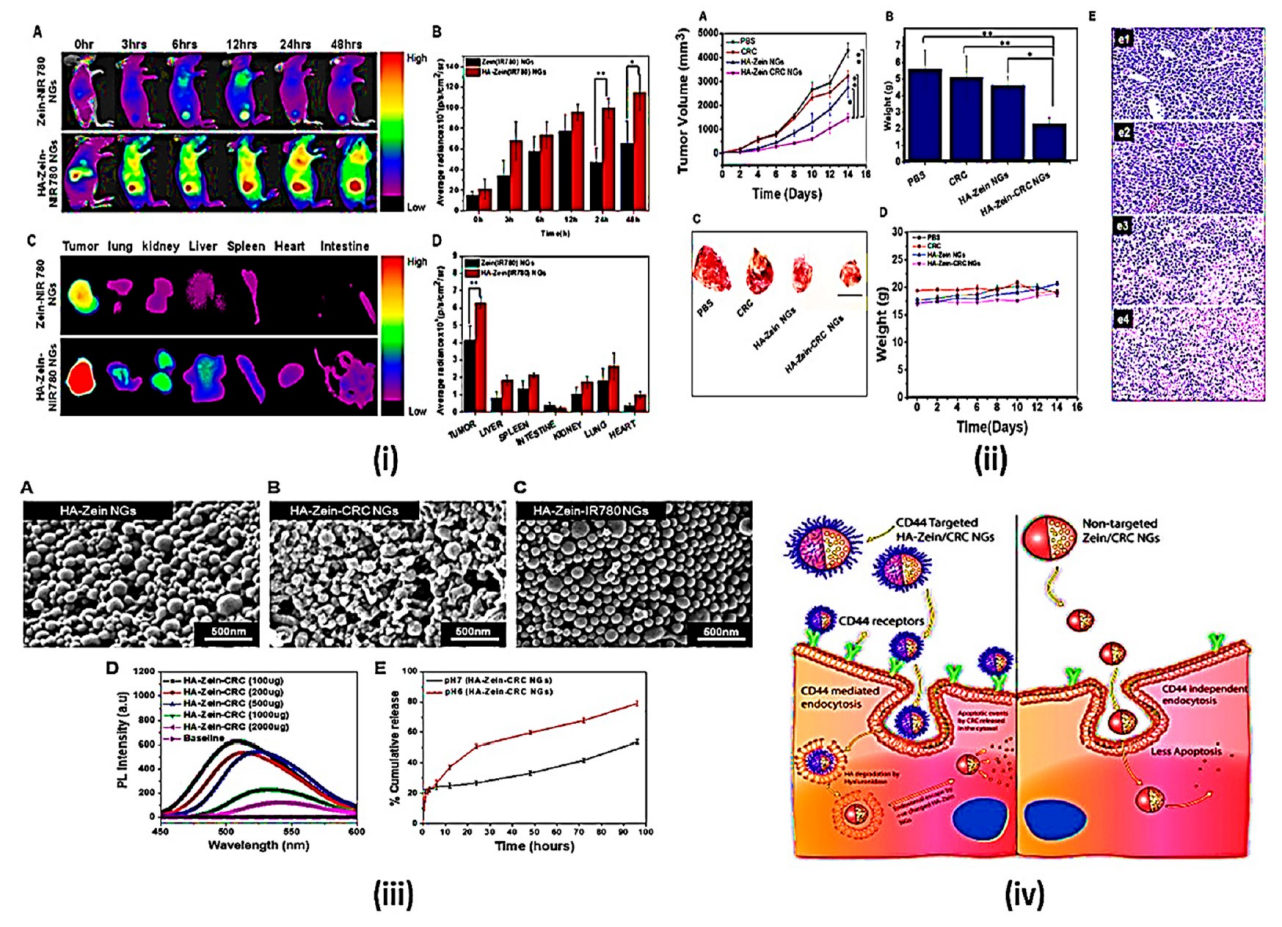
Delivery of curcumin through zein NPs upon functionalization with hyaluronic acid through targeted CD44. (i) *In vivo* imaging and biodistribution analysis of nude mice with CT26 tumors after the tail vein was injected with zein-IR780 and HA-zein-IR780 NGs. (A) Time-collapsed NIR fluorescence images of nude mice. The tumor is located on the thigh as a circle. (B) Qualitative analysis of the NIR fluorescence intensity of the tumor site at the indicated time points. (C) NIR fluorescence images of the major organs and tumors 72 h after injection of the NGs. (D) Accumulation of NGs at the tumors and organs by qualitative analysis with NIR fluorescence intensity. (ii) *In vivo* therapeutic effect of the curcumin encapsulated HA-zein NGs. (A) CT26 tumor growth curves for different treatment groups at specific time points. (B) Bar chart depicting the tumor weight after 14 days of injections. (C) Tumor images after treatment of each sample at 14 days (scale bar: 2 cm). (D) Body weight curves of the mice after the NG treatments. (E) Histological analysis of tumor tissues of the different sample treated groups such as (e1) PBS, (e2) CRC, (e3) HA-zein NGs, and (e4) HA-zein-CRC NGs, respectively, where maximum cellular damage is observed with HA-zein-CRC NGs (*n* = 5, ±SD, *p < 0.05, **p < 0.01). (iii) SEM images of the (A) HA-zein NGs, (B) HA-zein CRC NGs, and (C) HA-zein-IR780 NGs. (D) Photoluminescence of the HA-zein CRC NGs with varying CRC amounts. (E) Cumulative release percent of CRC from the HA-zein CRC NGs in two different pH environments (pH 7.4 and pH 6, *n* = 3). (iv) Graphical model. Reproduced from ref (117). Copyright 2018 Elsevier.

Soek et al. cross-linked hyaluronic acids with zein NPs facilitate the targeted delivery of curcumin through zein nanogels via upregulated CD44 in both *in vivo* and *in vitro* studies.^117^ Thus, the preclinical studies showed that these novel HA-zein NGs would be highly beneficial in encapsulating hydrophobic drugs with improved pharmacokinetics, thereby enhancing the therapeutic outcomes (Figure 12). Future studies targeting resolving the commercialization of biomaterial for delivering anticancer compounds must be a matter of focus.

So zein NPs are used for cancer treatment; various researchers did different *in vivo* and *in vitro* tests to check the drug potentials, and a different technique was also done for the characterization of the NP, which made it susceptible to cancer treatment.

For the liposomal attachment point of view, Lee et al. used zein phosphatidylcholine hybrid NPs enclosed with indocyanine green for cancer treatment. PC-NP shows excellent success in stabilizing ICG by embedding it in liposomes. ICG was obtained in Z/PC-NP without affecting the colloidal stability of the Z/PC-NP. Z/PC-NP stopped ICG depletion successfully, and the phototoxicity of ICG contained in Z/PC-NP was 2-fold more significant than that of PC-NP, whereas Z/PC-NP preserved its photo-cytotoxicity more than PC-NP. Hence Z/PC-NP is used for the treatment of cancers.^118^

**Figure 13:**
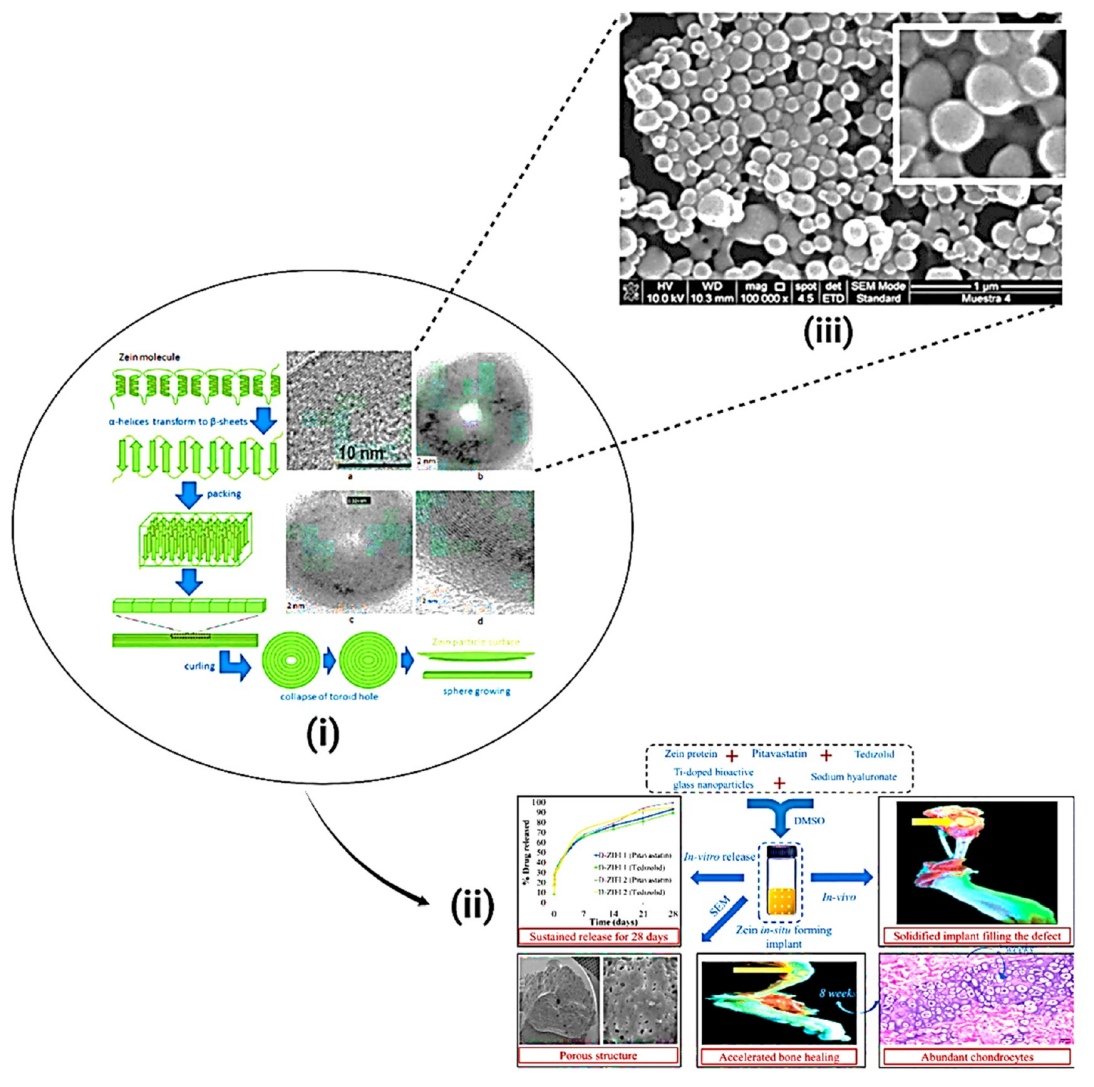
(i) Possible mechanism for zein self-assembly from single molecules to nanospheres: (TEM image (A)) α-helices in the original zein solution transformed into β-sheet strands; (TEM image (B)) antiparallel β-sheets packed side by side forming a long ribbon, driven by hydrophobic interactions; the ribbon curled into a toroid or ring; (TEM image (C)) the center hole of the toroid was closed; and (TEM image (D)) disks enlarged by the addition of new β-sheet strands.^121^ (ii) Schematic model depicting the role of drug delivery mediated by zein NPs. Reproduced with CC-BY license from ref (122). Copyright 2022 MDPI. (iii) Scanning electron microscopy (SEM) microphotograph of resveratrol-loaded zein NPs. The bar indicates the resolution (1 μm). The white box delimits a magnified area. Reproduced from ref (123). Copyright 2015 ACS.

In medical and biological delivery applications, it has been proven that zein is a biocompatible material. Zein could be used noninterferingly, such as in photosensitizer therapy, for killing cancer cells.^38,119^ Therefore, a unique strategy to treat gene-related chronic illnesses involves modifying the functions of the zein protein surface to target cellular or subcellular areas primarily. To increase the biomedical uses of this low-cost plant protein, it may be inferred that desired surface modification of the protein’s surface with a highly biomaterial synthetic or biological polymer is a possible tool that should be explored. This part summarizes the oncological application of bio-based zein nanomaterial with the structure, property, and physical performance of the drug delivery system.^119^ The challenges and perspectives of bio-based zein nanomaterials in oncotherapy will be discussed in the future. Hence, the current status of zein nanosystems and existent challenges make bio-substance-based NPs have a substantial effect on clinical application (Figure 13).

## 6.0 Challenges and Future Outlook

Taking a medicinal product from the lab to the patient’s bedside depends on several factors, including cost-effectiveness, safety, and biocompatibility.^124−128^ Some currently available materials have received widespread acceptance for most therapeutic advantages. Because of this, only a tiny number of nanomedicine products are general and are approved for use in clinical settings.^129−131^ Zein has certain unique benefits, including FDA “GRAS” designation, cost savings, biocompatibility, and medicinal features, including adhesiveness and ease of forming a vehicle in the right shape and size due to its soft and pliable nature.^42^ Immunogenicity may, however, be a limiting issue for its widespread application as a protein nanocarrier. Zein NPs’ immunological response will still need to be evaluated concerning size and dosage.^42,132,133^

As a plant protein, zein’s characteristics are influenced by its composition and the presence of other substances during its extraction and purification.^42^ Zein is a resource, but it is yet unknown how its extraction affects its medicinal, physiochemical, and therapeutic qualities.^47,87,95,134,135^ Zein’s brick-like structural properties and higher concentration of hydrophobic amino acids make it possible to load hydrophobic medicinal compounds. Hydrophilic polymers like PEG can alter micellar structures to load hydrophobic and hydrophilic medicines by conjugating with zein.^136^ A further viable option for more significant drug loading and controlled release is the direct conjugation of medication with zein to improve pharmacokinetics and therapy. Zein may increase the stability of other nanocarrier systems, making this a viable strategy for delivering therapeutic proteins and drugs sensitive to pH and enzymes.^137−140^

**Figure 14:**
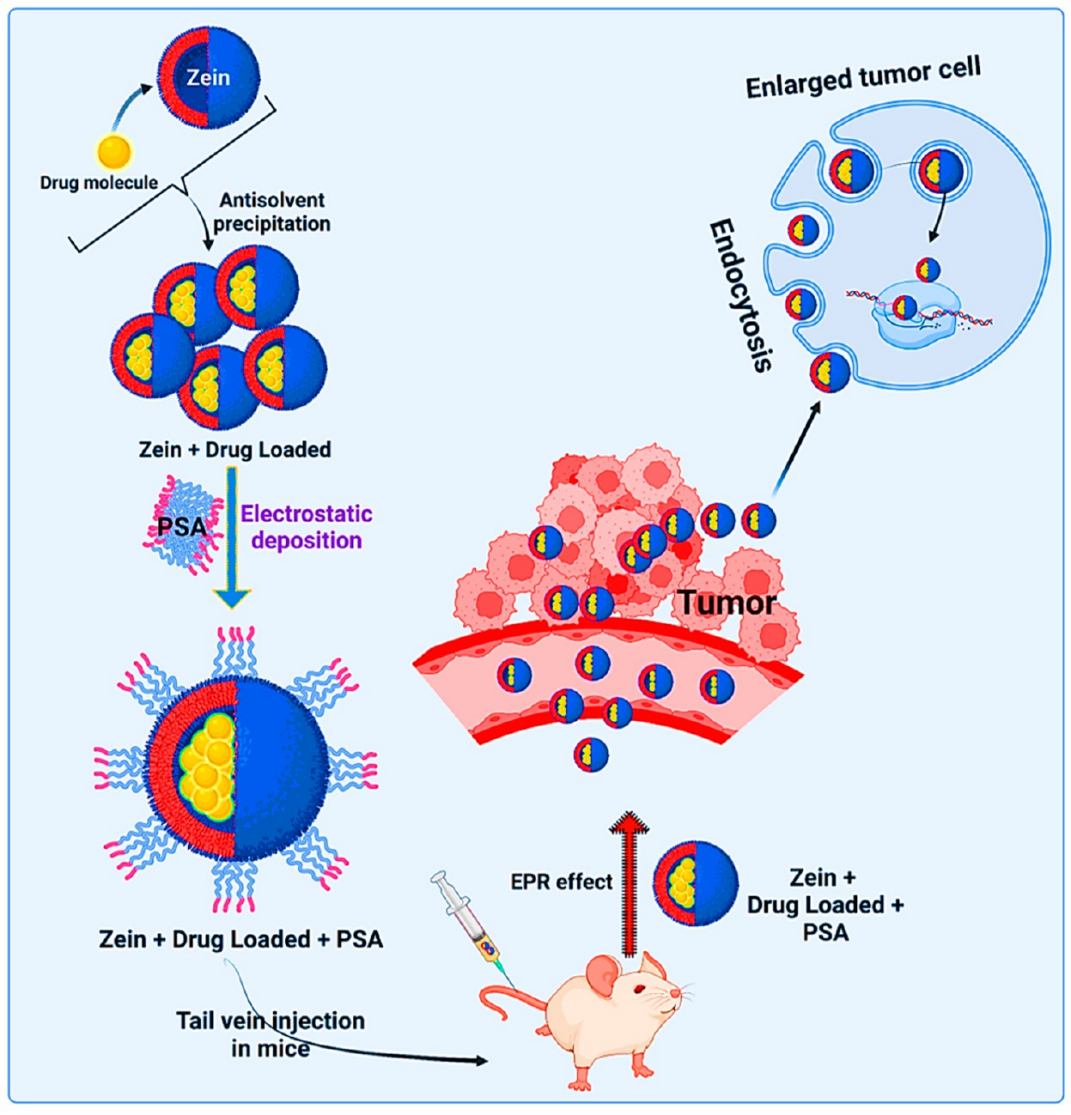
A graphical model depicting the projection of zein NPs loaded with a drug with electrostatic deposition for targeting enlarged tumor cells for assisted cancer drug delivery in precision medicine. The comparative analysis between zein nanoparticles and traditional methods in cancer therapy highlights several key distinctions and advantages. Traditional cancer treatment methods, such as chemotherapy and radiation, are often associated with systemic toxicity and nonspecific targeting, leading to significant side effects. In contrast, zein nanoparticles offer a more targeted approach. Their biocompatibility and ability to encapsulate both hydrophilic and hydrophobic drugs allow for precise delivery of therapeutic agents directly to cancer cells, minimizing damage to healthy tissues. When comparing zein nanoparticles with traditional nanoparticles not utilizing zein, several differences become apparent. Traditional nanoparticles often rely on synthetic materials, which can raise concerns regarding toxicity and biodegradability. Zein nanoparticles, derived from natural corn protein, are inherently biodegradable and less toxic, making them a safer alternative for long-term treatment. Their unique molecular structure also provides superior stability and encapsulation efficiency, crucial for effective drug delivery. Furthermore, zein nanoparticles exhibit distinct advantages in terms of drug release kinetics. They can be engineered to release their payload in a controlled manner, ensuring sustained therapeutic levels at the target site. This contrasts with some traditional nanoparticles, which may release drugs too rapidly or inconsistently, leading to suboptimal treatment outcomes.

However, further research is required to realize this biomaterial’s full potential in tailored medicine and vaccine delivery.^49,141,142^ The ability to easily modify the surface provides an opportunity to employ it as a site-specific, tailored drug delivery vehicle. Zein biomaterial might potentially replace synthetic polymers, which are now used for this purpose, in the future intracellular delivery of therapeutic peptides and genetic materials. Zein biomaterial might potentially replace synthetic polymers, which are now used for this purpose, in the future intracellular delivery of therapeutic peptides and genetic materials. To create zein-based NPs for gene therapy, the precise delivery of genetic material such as DNA, siRNA, and oligonucleotides into intracellular compartments may be explored. Zein is a biodegradable polymer that shares specific characteristics with poly(lactic-*co*-glycolic acid); however, there has not been any comparison research published yet. Being a hydrophobic biomaterial, it may provide a tremendous oral delivery system for medications that are not very water-soluble.^90,143−145^ Zein’s ability to self-assemble is helpful for high drug loading, and it may be further investigated to create cutting-edge multifunctional micelles for chemotherapy and imaging (Figure 14).^55,111,146,147^ Zein films offer a biocompatible foundation with enough space for cell development, making it practical for tissue engineering.^148,149^

Thus, we expect this plant protein to play a substantial clinical role in biomedical and medication delivery technologies in the future. To create a novel nanomedicine with potent antioxidant properties, additional research will be carried out to (i) investigate the mechanisms underlying the synergistic pharmacological effects of zein, (ii) assess the results of this formulation following post-treatment of stressed cells, and (iii) coencapsulate two bioactive in the protein matrix.

The advantages of zein nanoparticles extend to their versatility in gene therapy. Unlike traditional methods, which may use viral vectors with inherent risks, zein nanoparticles provide a safer, nonviral means of delivering genetic material to cells. This aspect is particularly crucial for reducing the potential for immune responses and other complications associated with gene therapy. Zein nanoparticles represent a significant advancement over traditional cancer treatment methods and conventional nanoparticles. Their biocompatibility, enhanced targeting capabilities, controlled drug release, and safety profile in gene therapy underscore their potential in revolutionizing cancer treatment and offering a more effective and less invasive alternative to conventional therapies.

The challenges and future outlook of zein NPs in medical applications present an insightful and innovative discourse, which is pivotal for advancing this promising field. A primary challenge lies in the scalability of production. Developing cost-effective and efficient large-scale manufacturing processes for zein NPs is crucial to the transition from laboratory research to clinical and commercial use. Addressing this will require innovative engineering solutions and possibly new synthesis methods that maintain the quality and consistency of the nanoparticles. Another significant challenge is the stability and storage of the zein NPs. Ensuring that these nanoparticles retain their structural integrity and functional properties over time, especially under varying environmental conditions, is essential for their practical application. Research is needed to improve formulations and develop novel stabilizing agents that can extend the shelf life of zein NPs without compromising their biocompatibility or effectiveness. Controlled and targeted drug release remains a complex issue. While zein NPs show promise in targeted delivery, achieving precise control over where and when the drug is released in the body is an area ripe for innovation. This could involve exploring smart release systems that are responsive to specific physiological triggers, such as pH changes or enzymes specific to disease sites.

In the realm of gene therapy, addressing the efficiency of gene delivery and ensuring the safety of delivered genetic material are crucial challenges. Future research should focus on enhancing the transfection efficiency of zein NPs and ensuring that the genetic material remains intact and functional upon delivery.

The future outlook for zein NPs is undeniably optimistic, with potential breakthroughs on the horizon. Advancements in nanotechnology, bioengineering, and materials science could lead to novel applications of zein NPs beyond cancer treatment and gene therapy, such as in targeted drug delivery for neurodegenerative diseases or as carriers for vaccines. The integration of zein NPs with other emerging technologies like CRISPR gene editing or AI-driven drug discovery could further enhance their efficacy and application scope.

While challenges such as scalability, stability, controlled release, and efficiency in gene delivery present hurdles, ongoing research and innovation in the field of zein NPs hold immense promise. Overcoming these challenges will not only revolutionize the application of nanotechnology in medicine but also pave the way for new therapeutic strategies, ultimately contributing to better patient outcomes and more effective treatments.

## 7.0 Conclusion

In the realm of nanotechnology, zein NPs are emerging as a revolutionary approach to the fight against cancer. Their capacity to encapsulate a diverse array of bioactive substances makes them particularly suitable for dietary, pharmacological, and biological applications. This study has focused on creating cost-effective, innovative zein-based nanoformulations with broad applicability. We extensively characterized the structural properties, synthesis, and preparation methodologies of zein NPs. Their role in drug and gene delivery systems for anticancer therapy is noteworthy. Zein enhances antitumor efficacy while minimizing required dosages, thus reducing side effects. The development of various anticancer compounds employing zein systems targeting cancer cells through multiple mechanisms is a significant advancement. The success of co-delivery systems further underscores their potential in effective cancer treatment. Zein NPs excel in encapsulating both hydrophilic and lipophilic substances, providing efficient release within complex environments. The zein coating notably impacts the physicochemical properties of drugs, affecting the particle size and colloidal stability, which are crucial for cancer regression. Despite these advances, zein nanoparticles have several challenges. Scalability of production and the assurance of long-term stability are significant hurdles. The controlled release of encapsulated agents, a key feature for effective treatment, remains a complex issue, requiring further research. Additionally, understanding the biological interactions and potential toxicity of zein NPs in human systems is critical for their safe application.

The future outlook for zein nanoparticles in cancer therapy is optimistic but contingent on overcoming these challenges. Advancements in research and development, complemented by clinical trials, are essential. There is a need to optimize formulations for specific cancer types, improve targeting efficiency, and ensure compatibility with existing treatment methods. Addressing these challenges will be pivotal in harnessing the full potential of zein nanoparticles in cancer therapy, paving the way for more effective and safer treatment options.
